# Estramustine Phosphate Inhibits TGF-*β*-Induced Mouse Macrophage Migration and Urokinase-Type Plasminogen Activator Production

**DOI:** 10.1155/2018/3134102

**Published:** 2018-09-02

**Authors:** Sonja S. Mojsilovic, Slavko Mojsilovic, Suncica Bjelica, Juan F. Santibanez

**Affiliations:** ^1^Laboratory for Immunochemistry, Institute for Medical Research, University of Belgrade, Dr. Subotića 4, 11129 Belgrade, Serbia; ^2^Laboratory for Experimental Hematology and Stem Cells, Institute for Medical Research, University of Belgrade, Dr. Subotića 4, 11129 Belgrade, Serbia; ^3^Group for Molecular Oncology, Institute for Medical Research, University of Belgrade, Dr. Subotića 4, 11129 Belgrade, Serbia; ^4^Centro Integrativo de Biología y Química Aplicada (CIBQA), Universidad Bernardo O'Higgins, General Gana 1780, 8370854 Santiago, Chile

## Abstract

Transforming growth factor-beta (TGF-*β*) has been demonstrated as a key regulator of immune responses including monocyte/macrophage functions. TGF-*β* regulates macrophage cell migration and polarization, as well as it is shown to modulate macrophage urokinase-type plasminogen activator (uPA) production, which also contributes to macrophage chemotaxis and migration toward damaged or inflamed tissues. Microtubule (MT) cytoskeleton dynamic plays a key role during the cell motility, and any interference on the MT network profoundly affects cell migration. In this study, by using estramustine phosphate (EP), which modifies MT stability, we analysed whether tubulin cytoskeleton contributes to TGF-*β*-induced macrophage cell migration and uPA expression. We found out that, in the murine macrophage cell line RAW 264.7, EP at noncytotoxic concentrations inhibited cell migration and uPA expression induced by TGF-*β*. Moreover, EP greatly reduced the capacity of TGF-*β* to trigger the phosphorylation and activation of its downstream Smad3 effector. Furthermore, Smad3 activation seems to be critical for the increased cell motility. Thus, our data suggest that EP, by interfering with MT dynamics, inhibits TGF-*β*-induced RAW 264.7 cell migration paralleled with reduction of uPA induction, in part by disabling Smad3 activation by TGF-*β*.

## 1. Introduction

Transforming growth factor-*β* (TGF-*β*) plays multifunctional roles in the homeostasis of the immune system by acting as one of the most potent immunosuppressive factors during inflammation progression and resolution [1, 2]. For instance, TGF-*β* regulates both the adaptive and innate immune cell recruitment to the site of inflammation [3]. Actually, this cytokine has profound effects on innate immune cells including myeloid precursors, mast cells, myeloid-derived suppressor cells, dendritic cells, neutrophils, and monocytes/macrophages [4]. Namely, macrophages express a continuum phenotype from classical activated macrophages or M1, with proinflammatory and antitumor properties to alternative activated macrophages or M2, which possesses immunosuppressive, anti-inflammatory and protumoral characteristics [5]. TGF-*β* induces macrophage polarization toward M2 phenotype, which helps in the process of adaptive immune system suppression and tissue repair [6, 7]. Moreover, in tumor microenvironment, TGF-*β* provokes macrophage differentiation toward a tumor-associated macrophages- (TAM-) like (M2-like) phenotype [8]. Both M2 and/or TAM cells are major sources of proteolytic enzymes that contribute to extracellular matrix (ECM) reorganization and favour the invasion of neoplastic cells [9]. One of the most expressed macrophage-activated ECM-degrading proteases is urokinase-type plasminogen activator (uPA) [7]. uPA critically regulates monocyte/macrophage chemotaxis and migration, and it contributes to differentiation of monocytes into macrophages, participates in the induction of M2 phenotype, and seems to be essential for macrophage infiltration into tumor microenvironment [10–15].

Macrophages are highly motile cells that quickly migrate in the direction of a specific signal, and this is accompanied by changes in the cell body and dynamic cytoskeletal rearrangement [16]. Namely, the microtubules (MT) cytoskeleton network plays several key roles in macrophage cell function, including antigen presentation, phagocytosis, and migration [17, 18]. The interference in microtubule organizing centre and MT network highly affects macrophage motility and directional migration patterns [19]; as a consequence, MT cytoskeleton is a potential target in tumor chemotherapies.

Estramustine phosphate (EP), a nitrogen mustard derivative of estradiol-17*β*-phosphate, is an antineoplastic agent that suppresses the MT dynamics, functions as antimitotic agent, and may trigger cell death by disrupting the nuclear matrix. Specifically, EP in vitro weakly inhibits polymerization and induces depolymerization by interacting with microtubule-associated protein or by direct interaction with tubulin [20, 21]. Initially, EP was thought as a double alkylating and estrogenic agent, but although it reduces plasma testosterone levels, by suppression of the pituitary–gonadal axis as results of estrone and estradiol produced by hydrolysis of EP, the putative alkylating effects are disabled [22].

TGF-*β* mainly transduces intracellular signaling via phosphorylation and activation of Smad2/3 transcription factors. These Smads interact with Smad4, and then this complex is translocated to the nucleus to exert its function on gene expression [23]. Intriguingly, it has been described that Smads binding to MT may regulate TGF-*β* signaling [24], which suggests that MT may play a regulatory role in TGF-*β* intracellular signal activity.

We previously demonstrated that TGF-*β* induces uPA expression via activation of Smad3 signaling in murine macrophages [25], so regarding the importance of uPA and MT network on cell migration, here, we determine whether the chemotherapeutic approach targeting microtubule dynamic by EP can modify the macrophage cell responses to TGF-*β*. We found out that EP, at non-antiproliferative concentrations, strongly reduces the cell migration induced by TGF-*β*, probably by inhibiting Smad3 activation and subsequent blocking of TGF-*β*-induced uPA in murine macrophages.

## 2. Material and Methods

### 2.1. Cell Line and Culture Conditions

Mouse macrophage RAW 264.7 (ATCC TIB-71) cell line was kindly provided by Dr. Carmelo Bernabeu (CIB, CSIC, Madrid, Spain). Cells were cultured in RPMI containing 10% FBS supplemented with antibiotics (100 units/ml streptomycin and 100 units/ml penicillin) in a 5% CO_2_ at 37°C and in a humidified culture incubator.

### 2.2. Reagents

Recombinant TGF-*β*1 was from R&D (Minneapolis, MN). Anti phospho-Smad3 (pSmad3) antibodies were purchased from Calbiochem (Darmstadt, Germany). The anti-Smad2/3 (sc-8332) polyclonal antibody was purchased by Santa Cruz Biotechnology Inc., CA. *α*-Tubulin, appropriate secondary antibodies coupled to horseradish peroxidase, FITC or TRIC, Smad3 inhibitor SiS3, bovine plasminogen, and estramustine phosphate (EP) were purchased from Sigma-Aldrich (St. Louis, MO).

### 2.3. Proliferation Assay

To determine the RAW 264.7 cell proliferation rate, by the MMT assay, we followed the methods of Krstić et al. [26]. The following day, cells were treated by the indicated EP concentrations for additional 24 or 72 hours. At the end of this period, 3-(4,5-dimethylthiazol-2-yl)-2,5-diphenyltetrazolium bromide (MMT, Sigma-Aldrich) was added to each well at 0.5 mg/ml, and cells were incubated for two additional hours. The culture medium was then discarded, and the cell-precipitated formazan crystals were dissolved in isopropanol: DMSO (3 : 2). The absorbance was read at 630 nm.

### 2.4. Immunofluorescence Assay

Immunofluorescence analysis was used to detect alpha-tubulin and pSmad3 in RAW 264.7 cells. Briefly, 10^5^ cells were seeded per rounded coverslip in 24 well plates and allowed to grow until 50% of confluence. After, the indicated treatments cells were fixed with 4% paraformaldehyde in PBS and permeabilized with 0.2% Triton X-100 for 5 minutes. Cell monolayers were incubated with specific primary antibodies, followed by incubation with corresponding fluorescently labelled secondary antibodies and 1 *μ*g/ml of 4′,6-diamidine-2′-phenylindole dihydrochloride (DAPI), Sigma-Aldrich (St. Louis, MO). After mounting with DABCO-Mowiol, the samples were examined and photographed using an epi-fluorescence microscope. pSmad3 positive cells were quantified by counting the number of positive cells in at least 5 different fields for each experimental conditions.

### 2.5. Cell Migration Assays

To analyse cell migration, by in vitro wound healing and Boyden chamber-based cell migration assays, we followed the methods of Krstić et al. [26]. Briefly, 5 × 10^4^ cells/well were seeded in 24 well plates and allowed to grow until confluence. A scratch wound in the monolayer was made by a 200 *μ*l pipette tip. Cells were washed three times with PBS and allowed to migrate for additional 24 hours with the indicated treatments. Then, cells were fixed with ice-cold methanol and stained for 30 minutes with 0.1% crystal violet. Cell migration into the scratch area was photographed using an inverted light microscope and quantified by TScratch software (Computational Science and Engineering Laboratory, Swiss Federal Institute of Technology, ETH Zurich, Switzerland).

The migration capacity of RAW 264.7 cells was also evaluated in a Boyden chamber-based cell migration assay (Costar, Cambridge, MA) with 8.0 *μ*m pore polycarbonate filters (Collaborative Research, Bedford, MA). Briefly, RAW 264.7 were labelled with carboxyfluorescein succinimidyl ester (CFSE, Sigma-Aldrich), according to manufacturer's instructions, and seeded in the upper chamber (10^5^ cells per transwell) in 150 *μ*l of growth medium. Growth medium (0.5 ml), with or without 5 ng/ml of TGF-*β*, as a chemoattractant factor, and EP were added in the lower chamber. After 18 h, cells from the upper compartment were cleaned with a cotton swab to remove the nonmigrating cells. Cells attached to the bottom of transwells were fixed by immersing the transwells into 3.7% formaldehyde in PBS. After washing, transwells were turned upside down, mounted with a coverslip, and cells were observed using an epi-fluorescence microscope. Green labelled cells from each sample were counted using ImageJ software in eight random fields per transwell insert.

### 2.6. Urokinase-Type Plasminogen Activator Activity Assays

uPA activity was determined by the radial caseinolysis assay in the conditioned, serum-depleted media from indicated RAW 264.7 cell treatments. Protein-normalized conditioned medium aliquots (50 *μ*l) were added to the wells made in the 1% agarose gel containing 0.5% casein, 2 *μ*g/ml plasminogen, and 10 mM CaCl_2_. Diameters of clear areas were measured after incubation at 37°C for 24 h. uPA activity was estimated as fold increase compared with conditioned medium from untreated cells. The uPA degradation areas were quantified by densitometry, using NIH-ImageJ software [16].

Cell culture supernatants were analysed for the presence of secreted uPA also by the zymography assay. Briefly, 5 × 10^4^ cells/well were seeded in 24 well plates, cultured overnight, and washed three times with PBS; then 0.5 ml of serum-free culture medium was added for additional 24 h cultivation under indicated treatments. Aliquots of protein-normalized conditioned medium were subjected to 10% SDS-PAGE under nonreducing conditions. After being washed with 2.5% Triton X-100, the gels were placed on 1% agarose gels containing 0.5% casein and 2 *μ*g/ml plasminogen and incubated at 37°C for 24 h. uPA-dependent proteolysis was detected as a clear band in the agarose gel. The uPA bands were quantified by densitometry, using NIH-ImageJ software [27].

### 2.7. Western Blot Assay

To determine proteins by the western blot assay, we followed the methods of Krstić et al. [26]. Cells, cultivated in six well plates at density of 1.5 × 10^6^ cells/well, were lysed for 30 min at 4°C in 200 *μ*l of RIPA lysis buffer (1% NP-40, 0.5% sodium deoxycholate, 0.1% SDS, 150 mM NaCl, 50 mM Tris, pH 7.5, with 1 mM Na_3_VO_4_ and protease inhibitors). Equal amounts of proteins of each extract were separated by SDS-PAGE and transferred to PDFV membranes (AppliChem, Darmstadt, Germany). The membranes were blocked in 4% nonfat milk in PBS buffer containing 0.5% Tween 20. The membranes were then incubated with primary, and subsequently with horseradish peroxidase-conjugated secondary antibodies. Specific protein bands were visualized using the ECL reagent from Thermo Scientific (Rockford, USA). The western blot bands were quantified by densitometry, using NIH-ImageJ software.

### 2.8. Transfection and Luciferase Assays

The uPA promoter transcriptional activity was determined by using a p-4.8 murine uPA–Luc luciferase reporter plasmid (provided by Dr. Munoz-Canoves (CRG, Spain)) as described [27]. The reporter assay with TGF-*β*/Smad3 responsive promoter construct was performed using p (CAGA)_12_-Luc construct as described [28]. Firefly luciferase activity, determined following manufacture's recommendations (Promega, Adison, WI, USA), was normalized for *β*-galactosidase activity (Tropix, Bedford, MA, USA).

### 2.9. Statistical Analysis

Data are given as means (±SEM) from at least three independent experiments. Student's *t*-test was performed to evaluate the probability of significant differences among the samples with *p* < 0.05 (^∗^) and *p* < 0.005 (^∗∗^) considered significant.

## 3. Results

### 3.1. EP Cytotoxicity and RAW 264.7 Cells Cytoskeleton Effects

**Figure 1:**
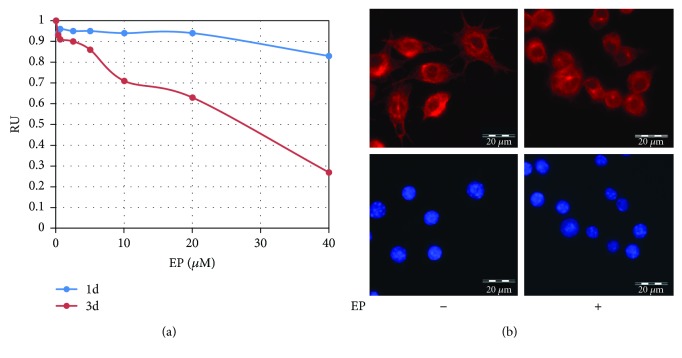
EP on RAW 264.7 cell proliferation and tubulin cytoskeleton. (a) RAW 264.7 cells were treated with increasing concentrations of EP for one or three days, and cell proliferation was determined in a triplicate experiment by the MTT assay. At day one, EP showed reduced capacity of inhibiting cell proliferation compared to three-day treatment. RU: relative units. (b) One-day EP treatment, at 10 *μ*M, modified tubulin cytoskeleton determined by immune fluorescence (red). Nuclei were counterstained with DAPI (blue). Representative microphotographs from three independent determinations are shown.

Due to the fact that EP has been demonstrated to interfere mitosis and trigger cell death [20, 21], we first analysed the effects of EP on RAW 264.7 cell proliferation. Cells were subjected to cell proliferation for 24 and 72 hours (Figure 1(a)). Clear reduction of cell proliferation is observed at 72 h of EP treatment, from 10% of inhibition at 2.5 *μ*M to 73% at 40 *μ*M. Meanwhile, at 24 h of EP treatment at 40 *μ*M, only 18% cell reduction was obtained. Since next experiments will be performed for a maximum of 24 h, we decided to use EP at 10 *μ*M to avoid proliferative interaction with the subsequent analyses. To confirm that EP 10 *μ*M affects tubulin cytoskeleton, the immunofluorescence assay was performed (Figure 1(b)). Epifluorescent imaging of *α*-tubulin revealed a disruption of the interphase microtubules in treated cells compared with control macrophages. Moreover, RAW 264.7 cells exhibited a spherical morphology in the presence of EP (data not shown).

### 3.2. EP Inhibits TGF-*β*-Induced Cell Migration

**Figure 2:**
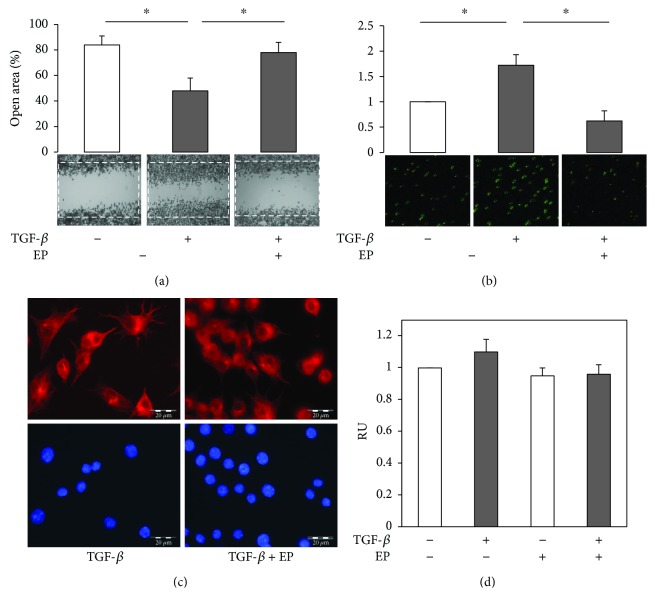
EP inhibits TGF-*β*-induced RAW 264.7 cell migration. (a) Cells were cultured until confluence, and a scratch at the centre of monolayer was made. Then, cells were treated as indicated for 24 hours, fixed and stained. 10 *μ*M EP significantly inhibits the closure of the wound induced by TGF-*β* 5 ng/ml. (b) RAW 264.7 cells were subjected to chemoattractant response to TGF-*β* 5 ng/ml by using the Boyden chamber-based assay. CFSE stained cells were allowed to migrate across the 8 *μ*m pore membrane for 18 hours. Microphotographs show fixed cells in the bottom of the membrane (green). EP 10 *μ*M significantly reduced TGF-*β* chemoattractant potency. (c) EP modified tubulin cytoskeleton in the presence of TGF-*β*. Cells were treated with TGF-*β* 5 ng/ml in the presence or absence of EP 10 *μ*M for 24 h. Then, cells were subjected to immunofluorescence analysis for tubulin cytoskeleton (red) and nuclei (blue). (d) Neither TGF-*β* nor EP modified RAW 264.7 cell proliferation, determined by the MTT assay. RU: relative units. Representative results from three independent experiments are shown. Significant difference between treatments by *t*-test: ^∗^ *p* < 0.05.

TGF-*β* has been demonstrated to induce macrophage cell migration toward the site of inflammation [3]. Next, we examined whether EP may interfere with macrophage motility determined by the wound healing assay. As observed in Figure 2(a), 18 h of TGF-*β* treatment enhances the capacity of RAW 264.7 cells to migrate into the wound in comparison with the control cells, while the presence of EP 10 *μ*M significantly abolished TGF-*β*-induced cell motility. EP also inhibited TGF-*β* chemoattractant function, since it reduced RAW 264.7 cell capacity to migrate through the 8 *μ*m pores to the bottom side of the membrane in a Boyden chamber-based cell migration assay (Figure 2(b)). The EP migration inhibition was related to changes in the RAW 264.7 cell tubulin cytoskeleton. As shown in Figure 2(c), EP modified the MT pattern compared with TGF-*β*-treated cells. The effect of EP on TGF-*β*-induced RAW 264.7 cells motility seems to be related to the changes in MT cytoskeleton and not to modifying cell proliferation. Neither TGF-*β* nor EP has shown to modify cell proliferation at the indicated experimental conditions (Figure 2(d)), nor have they had significant effects on the cell cycle (Supplementary Figure (available here)).

### 3.3. EP Inhibits TGF-*β*-Induced uPA Expression

**Figure 3:**
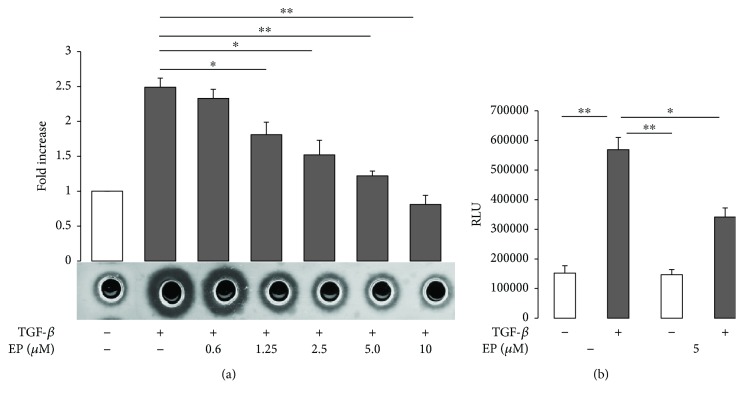
EP inhibits TGF-*β*-induced uPA production. (a) RAW 264.7 cells were cultivated with serum-free media for 24 h in the presence of TGF-*β* at 5 ng/ml and indicated EP concentrations. Then conditioned media were subjected to the radial caseinolysis assay. Degradation areas represent uPA activity. Increased EP concentration strongly inhibits the TGF-*β* capacity to induce uPA production. (b) EP inhibits TGF-*β*-induced uPA promoter transactivation. RAW 264.7 cells were transfected with a p-4.8 murine uPA–Luc luciferase reporter plasmid. Then, cells were subjected to TGF-*β* 5 ng/ml treatment in the presence or absence of EP 10 *μ*M. RLU: relative luciferase units. Representative results from three independent experiments are shown. Significant difference between treatments by *t*-test: ^∗^ *p* < 0.05 and ^∗∗^ *p* < 0.005.

Previous reports indicate that TGF-*β* is a potent inductor of uPA expression in macrophages and uPA contributes to macrophage cell migration [14, 15, 25]. We analysed whether EP inhibits the capacity of TGF-*β* to induce uPA in RAW 264.7 cells. The radial caseinolysis assay revealed that EP inhibited TGF-*β*-induced uPA secretion of RAW 264.7 cells, which is evident between 2.5 to 10 *μ*M of EP (Figure 3(a)). EP inhibition of TGF-*β*-induced uPA secretion seems to affect uPA expression at the transcriptional level, since the drug strongly inhibited the capacity of TGF-*β* to enhance the transactivation of the uPA promoter. Thus, these data suggested that EP, in part, reduced TGF-*β* enhancement of RAW 264.7 cell migration by blocking uPA expression incremented by the growth factor.

### 3.4. EP Inhibits Smad3 Activation by TGF-*β* Paralleled with Smad3-Dependant Migration and uPA Expression

**Figure 4:**
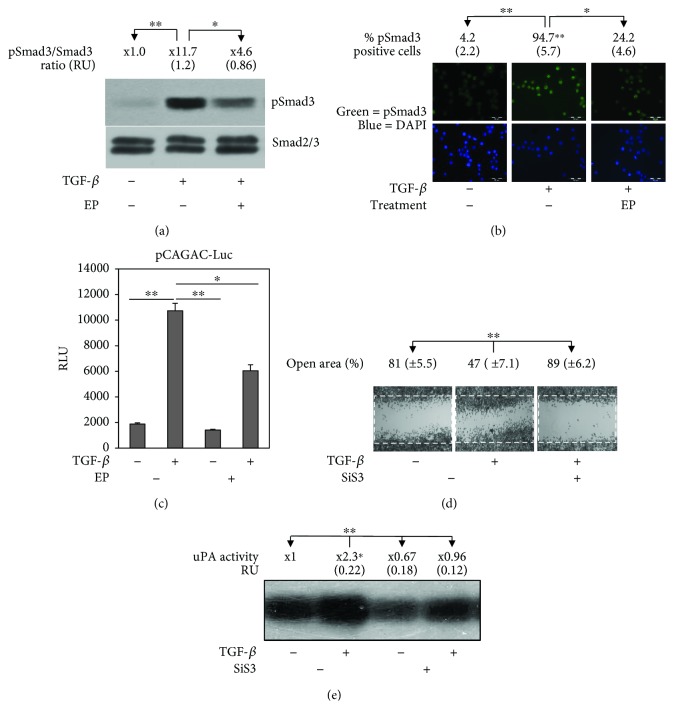
EP inhibits TGF-*β*-induced Smad3 activation. (a) TGF-*β* 5 ng/ml was used to one-hour stimulation of RAW 264.7 cells in the presence or absence of EP 10 *μ*M. Then, samples were subjected to the WB assay to determine Smad3 phosphorylation levels. EP reduced the capacity of TGF-*β* to increase phospho-Smad3 levels. (b) Nuclear phospho-Smad3 localization. Cells were treated for one hour with TGF-*β* 5 ng/ml in cotreatment with EP 10 *μ*M. Then cells were subjected to the immunofluorescence assay for Smad3 (green) and nuclei DAPI stains (blue). EP reduced the TGF-*β*-induced pSmad3 nuclear levels as is indicated by the number of pSmad3 positive cells. (c) EP inhibits Smad3 responsive p (CAGA)_12_-Luc construct transactivation. Transfected cells were treated with TGF-*β* 5 ng/ml with or without EP 10 *μ*M for 24 h. Then, luciferase activity was determined. RLU: relative luciferase units. (d) Direct Smad3 inhibition by using SiS3 reduces TGF-*β*-induced cell migration, determined by the wound healing assay. (e) Smad3 inhibition decreases TGF-*β*-induced uPA secreted activity, determined by the zymography assay. Representative results from three independent experiments are shown. Significant difference between treatments by *t*-test: ^∗^ *p* < 0.05 and ^∗∗^ *p* < 0.005.

We previously demonstrated that TGF-*β* induces uPA expression by Smad3 signaling [25], and Smads have been reported to be associated with MT cytoskeleton [24]. Subsequently, we examined whether EP effect on the tubulin network may modify Smad3 activation by TGF-*β*. As shown in Figure 4(a), the western blot assay revealed that EP strongly inhibits the capacity of TGF-*β* to induce Smad3 phosphorylation, which is accompanied with a decrease of the nuclear phospho-Smad3 presence after TGF-*β* and EP cotreatment (Figure 4(b)). This diminution in Smad3 phosphorylation and nuclear translocation was paralleled with EP inhibition of TGF-*β*-induced transactivation of the Smad3 reporter (Figure 4(c)), which confirms that EP, by interfering on the MT cytoskeleton network, inhibits the capacity of TGF-*β* to activate Smad3 signaling. The importance of Smad3 on TGF-*β*-induced RAW cell migration was demonstrated by using the chemical Smad3 inhibitor SiS3.  Figure 4(d) shows that SiS3 at 5 *μ*M reduced the capacity of TGF-*β* to increment cell migration analysed by the wound healing assay. Moreover, SiS3 is also able to reduce TGF-*β*-induced uPA production as revealed by zymography analysis (Figure 4(e)). These data suggest that EP, via its interference on MT cytoskeleton, inhibits TGF-*β*-induced RAW 264.7 cells motility and uPA expression by reducing Smad3 activation.

## 4. Discussion

TGF-*β*, in the immune system, has been demonstrated to profoundly modulate monocyte/macrophage functions [3]. TGF-*β* promotes recruitment of monocytes, and it may promote monocyte to macrophage differentiation [29]. For instance, TGF-*β* promotes macrophage polarization toward an M2-versus-M1 phenotype, which further promotes TGF-*β* production and deepens TGF-*β*-induced immunosuppression [4, 30, 31]. Moreover, in tumor microenvironment, TGF-*β* induces macrophage differentiation toward a TAM-like (M2-like) phenotype [23], which further supports tumor growth. Actually, TAMs seem to be crucial and the most abundant immune cell components in tumor microenvironment [32]. Furthermore, in cancer, the TAM level is strongly associated with poor prognosis and survival, which made these cells attractive targets for chemotherapeutic interventions [33]. In this study, by using RAW 264.7 murine macrophage cell line, we investigated the effect of EP, a widely antineoplastic agent that interferes with MT dynamics, on TGF-*β*-induced cell migration and uPA expression.

TGF-*β* is a pleotropic factor that possesses a dual role in tumorigenesis, since it acts as tumor suppressor at early steps of tumor progression, while it is a prometastatic cytokine in aggressive cancer stages [34]. Moreover, excessive TGF-*β* levels recruit monocytes within tumor microenvironment and may promote the alternative activation of M2 macrophages in detriment of M1 polarization, thereby promoting tumor progression [8]. The recruitment of monocytes-macrophages to tumor stroma requires intense acquisition of motile cell phenotype. Cell migration implies the contribution of regulatory pathways spatially and temporary engaged with cytoskeleton plasticity and reorganization [16, 35]. Actually, tubulin cytoskeleton is a key for the establishment of cell polarity, cell extensions, and cell migration. Furthermore, the microtubule network finely coordinates with actin filaments for proper cell migration [36, 37]. To determine the role of microtubule in macrophage cell migration, we used EP as a microtubule dynamic interfering agent. Namely, in vitro analysis indicated that estramustine and EP bind to tubulin dimers and to microtubule-associated proteins, inhibiting microtubule assembly and suppressing the dynamic instability of individual MAP-free microtubules [20]. Here, we used noncytotoxic EP concentration (10 *μ*M) for 24 hours of treatment, which effectively modified the RAW 264.7 cell microtubule network (Figure 1), to determine its effect on TGF-*β* cell response. It has been demonstrated that TGF-*β* induces monocyte/macrophage cell migration to the site of injury [37, 38]. Our results also indicated in vitro TGF-*β* increased cell migration in wound healing and Boyden chamber-based cell migration assays (Figure 2), whose effects were strongly inhibited by EP. This inhibition implicated the EP-dependent changes of the MT network but without effect on cell proliferation, since neither TGF-*β* alone nor in the mix with EP modified cell growth in our experimental conditions (Figure 2(d) and Supplementary Figure). These results suggested that EP treatment interfered with the stimulation of cell migration and the chemoattractant activity of TGF-*β* on murine macrophages.

The cell capacity to breakdown the ECM is also one of the main mechanisms implicated in cell migration [39]. In that sense, macrophages highly produce several ECM-degrading proteases, such as matrix metalloproteases, cathepsins, and uPA, which are implicated in both macrophage migration and in enhancement of migration of cancer cells [40]. Specifically, uPA has been demonstrated to contribute to macrophage migration and invasion. uPA can stimulate macrophage migration, and the silence of uPA or its receptor uPAR impairs in vivo macrophage tumor infiltration as well as in vitro Matrigel invasion [11, 14]. Moreover, uPA is a key mediator of macrophage 3D invasion and ECM remodelling [15]. TGF-*β* is a potent inductor of the uPA gene transcription; it increases uPA-mRNA stability and activates macrophage uPA expression [41, 42]. Our results indicate that EP strongly inhibited TGF-*β* capacity to stimulate uPA expression in RAW 264.7 cells (Figure 3). This effect seems to involve the uPA transcriptional gene inhibition mechanism, since EP decreased TGF-*β*-induced uPA promoter transactivation, which may indicate that EP by modulating MT network dynamics may interfere with TGF-*β* intracellular signaling propagation to the nucleus. Also, these data allowed us in part to exclude the involvement of EP on uPA transport and exocytosis, since MT are highly implicated in secretory pathways as well [43].

We previously investigated the intracellular signal transduction implicated in the capacity of TGF-*β* to induce uPA expression in RAW 264.7 cells. We demonstrated that Smad3, a member of the canonical TGF-*β* signaling, contributes to the increased production of uPA [25]. Intriguingly, TGF-*β*–Smad signaling has been shown to be regulated by tubulin cytoskeleton. Dong et al. [24] demonstrated that the total disruption of the MT network increased cell TGF-*β* responses, thus indicating MT as a negative regulator of TGF-*β*–Smads signaling. We found out that EP inhibited TGF-*β*-induced Smad3 phosphorylation, it reduced phospho-Smad3 nuclear localization, and it decreased Smad3 transcriptional activity. In addition and as we expected, the inhibition of Smad3 signaling by using the chemical inhibitor SiS3 [44] greatly reduced TGF-*β*-induced macrophage migration and uPA production (Figure 4 and [25]). MT undergoes stochastic changes between polymerization and depolymerization, namely, dynamic instability [45], and any interference with this dynamic may affect the signal propagation from the extracellular to the nucleus. Estramustine, at the range of concentration used in this study, has been demonstrated to suppress the dynamic instability by incrementing tubulin acetylation that is implicated in MT stabilization [21]. So, we can speculate that EP, by stabilizing MT, may reduce the release of Smad3 from cytoskeleton to interact with the TGF-*β* type I receptor (T *β* RI), therefore inhibiting Smad3 phosphorylation. Furthermore, it has been demonstrated that TGF-*β* induces T *β* RI interaction with microtubules in a Smad7/adenomatous polyposis coli-dependent fashion to regulate migratory responses [46], which also can be interfered by EP effect on MT. Further experiments are necessary to challenge whether EP interferes with T*β*RI and Smad3 interaction in RAW 264.7 cells.

EP estrogenic effects may also be involved on the regulation of TGF-*β* signaling, either by the estradiol-17*β* part of EP or by estradiol releasing as results of EP hydrolysis [22]. In this sense, estradiol by inhibiting Smad and RHO signaling reduces the capacity of TGF-*β* to induce fibroblast activation [47]. In addition, estradiol inhibits TGF-*β* induction of extracellular matrix in human and rat mesangial cells [48]. Further experiments are necessary to determine whether estrogenic EP properties, beyond its tubulin cytoskeleton modulation capacity, may affect TGF-*β* signal transduction in macrophages.

Macrophages play important roles in the tumor progression [49], and it is critical to elucidate the EP in vivo effects as well as in primary macrophages such as mouse peritoneal macrophages or bone marrow macrophages. In this study, we used immortalized macrophage cell line RAW 264.7, which limits the scope of our results. Further extensive studies in vivo, in primary healthy and tumor-associated macrophages, are necessary to validate and fully understand EP modulation of TGF-*β* signaling and its effects in macrophage biology. Therefore, due to the importance of TAMs in tumor progression, it will be worthy to further examine if EP may reeducate macrophages from protumorigenic M2 phenotype to M1 activation, which can suppress tumor growth and angiogenesis [50]. The MT stabilization by using anti-MT drugs may promote macrophage polarization toward M1 phenotype [51], which suggests MT dynamics modulation as an emerging attractive strategy for therapeutic intervention of protumorigenic macrophage infiltration within tumor microenvironment.

## 5. Conclusions

The obtained results indicate that EP reduced TGF-*β*-induced uPA expression and cell migration, in part, by inhibiting Smad3 activation in murine macrophage RAW 264.7 cell line.

## Data Availability

The experimental data used to support the findings of this study are available from the corresponding author upon request.

## Supplementary Materials

Supplementary Materials RAW cells were treated for 24 hours with TGF-*β* 5 ng/ml with or without EP 10 *μ*M. Then, cells were subjected to flow cytometer cell cycle analysis. Briefly, treated cells were 3x PBS washed and fixed with 70% cold ethanol. Then, cells were treated with RNAse A (10 *μ*M/ml)/PBS for 30 minutes and stained with propidium iodide (5 *μ*g/ml). The figure shows that cell treatments did not modify the cell frequency (%) in the different cell cycle phases.
