# Supplementary material for: Estramustine Phosphate Inhibits TGF-β-Induced Mouse Macrophage Migration and Urokinase-Type Plasminogen Activator Production
Source: Anal Cell Pathol (Amst). 2018 Sep 2;2018:3134102. doi: 10.1155/2018/3134102 (PMC6139214; doi:10.1155/2018/3134102)
Supplement: Supplementary Materials — RAW cells were treated for 24 hours with TGF-β 5 ng/ml with or without EP 10 μM. Then, cells were subjected to flow cytometer cell cycle analysis. Briefly, treated cells were 3x PBS washed and fixed with 70% cold ethanol. Then, cells were treated with RNAse A (10 μM/ml)/PBS for 30 minutes and stained with propidium iodide (5 μg/ml). The figure shows that cell treatments did not modify the cell frequency (%) in the different cell cycle phases. [file 3134102.f1.pdf]

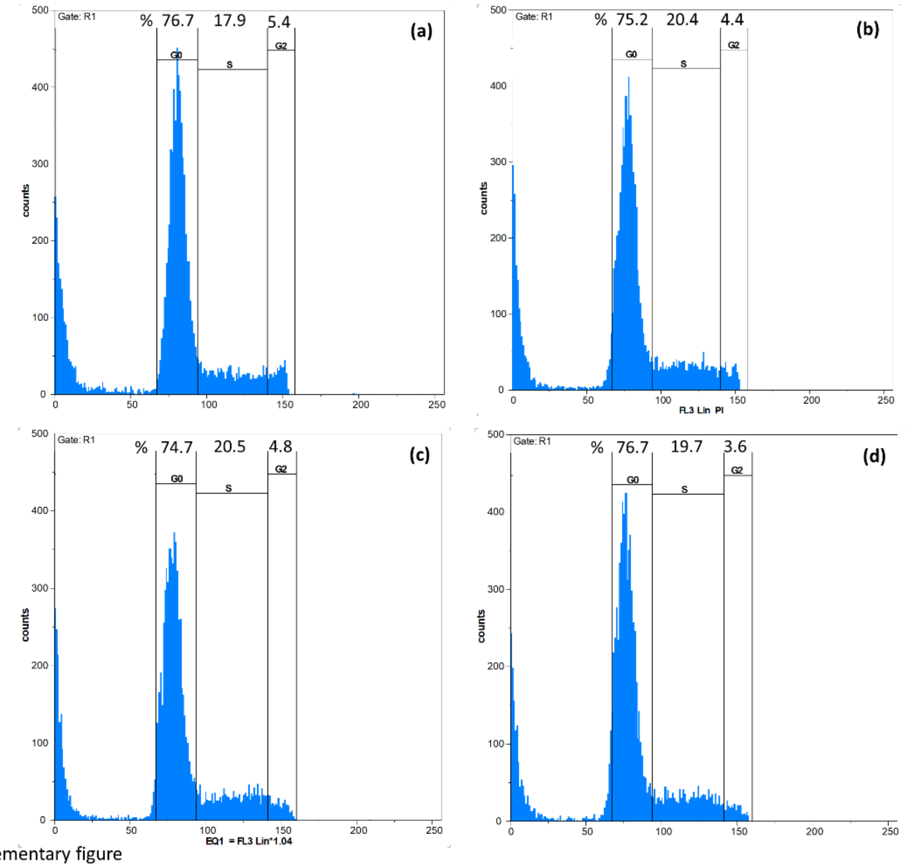

Supplementary figure

Supplementary Figure.

RAW cells were 24 hour treated with TGF-β 5 ng/ml with or without EP 10 μM. Then, cells were subjected to flow cytometer cell cycle analysis. Briefly, treated cells were 3X PBS washed and fixed with 70% cold ethanol. Then, cells were treated with RNAse A (10 μM/ml)/PBS for 30 minutes and stained with Propidium Iodide (5 μg/ml). Figure shows that cell treatments did not modify the cell frequency (%) in the different cell cycle phases.
